# Inequalities of quality of life in unemployed young adults: A population-based questionnaire study

**DOI:** 10.1186/1475-9276-6-1

**Published:** 2007-03-21

**Authors:** Lars Axelsson, Ingemar H Andersson, Lena Edén, Göran Ejlertsson

**Affiliations:** 1 Department of Health Sciences, Kristianstad University, S-291 88 Kristianstad, Sweden; 2 Department of Community Medicine, Lund University, S-205 02 Malmö, Sweden

## Abstract

**Background:**

It is well known that unemployment is a great problem both to the exposed individual and to the whole society. Unemployment is reported as more common among young people compared to the general level of unemployment. Inequity in health status and life-satisfaction is related to unemployment. The purpose of this population-based study was to describe QOL among unemployed young people compared to those who are not unemployed, and to analyse variables related to QOL for the respective groups.

**Methods:**

The sample consisted of 264 young unemployed individuals and 528 working or studying individuals as a reference group. They all received a questionnaire about civil status, educational level, immigration, employment status, self-reported health, self-esteem, social support, social network, spare time, dwelling, economy and personal characteristics. The response rate was 72%. The significance of differences between proportions was tested by Fisher's exact test or by χ^2 ^test. Multivariate analysis was carried out by means of a logistic regression model.

**Results:**

Our results balance the predominant picture of youth unemployment as a principally negative experience. Although the unemployed reported lower levels of QOL than the reference group, a majority of unemployed young adults reported good QOL, and 24% even experienced higher QOL after being unemployed. Positive QOL related not only to good health, but also to high self-esteem, satisfaction with spare time and broad latitude for decision-making.

**Conclusion:**

Even if QOL is good among a majority of unemployed young adults, inequalities in QOL were demonstrated. To create more equity in health, individuals who report reduced subjective health, especially anxiety need extra attention and support. Efforts should aim at empowering unemployed young adults by identifying their concerns and resources, and by creating individual programmes in relation not only to education and work, but also to personal development.

## Background

Several studies have reported that unemployment is associated with adverse health [1,2] and low life satisfaction [3]. A recent study in eleven states in the European Union (EU) showed that unemployment led to lower levels of life satisfaction in all the countries studied [4].

In most of the OECD countries unemployment among young people is much higher than the general level, often two and sometimes three times higher [5]. However, the unemployment rates for young people vary greatly between the European countries [6]. Unemployment is a chronic problem in most Western countries [7]. Unemployment creates great inequalities in health for considerable groups of young adults and it is associated with deterioration in health behaviour among young people [8]. It has recently been shown that health problems among unemployed adolescents can contribute to adult health problems [9].

All the Scandinavian countries have a rather generous level of unemployment benefits compared with the overall EU average. Still, economic deprivation was found to be the single factor with the strongest connection to mental health problems among unemployed young adults in the Nordic countries [10].

One of the most frequently cited theories concerning unemployment and ill health is Jahoda's functional or deprivation theory [11]. This theory is based on the needs other than economic needs – i.e. latent functions such as time structure, contact with others, individual goals, personal status and identity, and activity – that a job should fulfil in order to be a good one.

Jahoda [11] discussed the effects of unemployment starting from what characterises the job. Warr [12] has developed her theory, but he does not use a clear distinction between work and unemployment in his theory; instead he tried to highlight the experiences gained from both work and unemployment. Ezzy [13] suggested, in contrast to Jahoda and Warr, that decreased mental health when unemployed is a product of failure to find a meaningful existence. Concerning Jahoda's theory, Ezzy argued that the functional approach does not explain variations in the experiences and the effects of unemployment. However, Nordenmark [14] found support for the latent functions derived from Jahoda's [11] theory, but he also stressed that the psychosocial meaning of paid work varies among the unemployed.

Ezzy [13] proposed a theory according to which transition to unemployment can be seen as a status passage. According to him, status passages can be divided into two basic types: divestment passages and integrative passages, the former emphasising separation from a status. In such a status passage the individual moves to another part of a social structure, which involves benefits or losses for the individual. It can mean changed identity and self-esteem or changed behaviour. The unemployed person may end up in an existential vacuum caused by the divestment passages, resulting in damaged mental health and self-esteem.

According to Petito and Cummins [15], subjective QOL among adults is remarkably stable on a population basis, while QOL is lower and unstable in adolescence. Thus it seems important to study the development of QOL during the transition from adolescence to early adulthood.

The concept of QOL is rooted in welfare research and can be used at both the societal and the individual level. There is no consensus about the meaning of QOL and thus no generally accepted definition, but it is a common view that welfare is a broader concept that includes QOL [16]. Measuring QOL at the societal level can, for instance, include variables such as level of living, income distribution, suicide rates and health status in the population. At the individual level QOL can refer to variables such as health status, standard of living, work and housing conditions or experienced happiness, well-being and life satisfaction.

According to Ventegodt [17], factors such as relations with oneself, partner or children, satisfaction with sex life and social network seem much more important to the individual's QOL than employment status. It is also known that subjective health predicts QOL [18]. The high unemployment rates in the 1990s provided new groups of unemployed in industrial countries, and new research is needed to focus on the QOL of the large groups of unemployed young people from the 1990s on.

Since there is a lack of a generally accepted definition of QOL, it is defined operationally in different studies. Farquhar [19] describes four main types of definitions of QOL as a taxonomy: global, component, focused and combination definitions of QOL. Global definitions of QOL are to be seen as all-encompassing, permitting the individual to decide what components to include without defining them explicitly. This study deals with subjective global QOL, reported as self-assessed life situation by the individual.

The purpose of this population-based study was to describe QOL among unemployed young people compared to those who are not unemployed, and to analyse variables related to QOL for the respective groups. Knowledge about factors that have an impact on QOL among people can be of significance in understanding and defining circumstances that influence their life situation. This understanding can make it possible to increase equity in health and QOL among young adults.

## Methods

The study was performed in Kristianstad municipality in southern Sweden, with about 70000 inhabitants. In September 1998, a questionnaire was sent to all 264 individuals aged 20–25 who were registered at the employment agency as unemployed at that time, and had been unemployed for at least three months. A corresponding questionnaire was sent to 528 individuals of the same age group, randomly selected from the population register and not registered at the employment agency.

The questionnaire included questions about household composition, educational level, immigration, employment status, self-reported health, self-esteem, social support, social network, spare time, dwelling, economy and personal characteristics. The questionnaire was tested in a pilot study (n = 11) and adjusted before use.

The response rate was 73% for the unemployed group (192 out of 264), and 71% for the reference group (377 out of 528). Of the 192 participants registered as unemployed, 158 were actually unemployed for at least three months according to the answers in the questionnaire, and therefore designated as *unemployed*. Of these, 39% had never had an ordinary job. For the remaining 34 the answers in the questionnaire differed from the registration information, so they were not classified as unemployed for at least three months.

Among the 377 in the reference group, 20 were unemployed or had not declared their employment status, so they were not classified as references. The 357 *in the reference group *consisted of those who were employed (199), studying (149), and nine others (five on parental leave, two trainees and two doing military service). The study design, measurements and definitions have been described in detail previously [8].

Among the unemployed 44% were males and 56% females, and in the reference group 47% were males and 53% females. The educational level was homogeneous, with 76% of the young adults having completed upper secondary school. In Sweden, most adolescents finish their upper secondary school at the age of 19, and the subjects in this study were in the transition to the labour market as unemployed, students or employed young adults.

A non-response analysis was performed as telephone interviews among a random sample of the non-responders. Seventeen among the unemployed and 38 in the reference group were asked a few questions from the questionnaire (questions about education, employment status and immigration). With respect to these variables and sex, the differences between responders and non-responders were small in both the unemployed and the reference group.

In the present study the variables household composition, educational level, QOL, subjective health, self-esteem, mental health and very good social support from at least one of the persons – father, mother, partner/spouse/cohabitee (designated partner in results), any relative or any friend – were compared between the employed and those who were studying. One significant difference was found: more employed females were married/cohabited and lived with children compared to student females. The other variables did not differ between those working and those studying. It therefore seemed reasonable to combine employed and students into one control group.

### Measurements and definitions

*Quality of life *(QOL) is here referred to as the individuals' evaluation of their life contents, i.e. their global QOL, a definition in accordance with how other authors define QOL [16,18,20]. In this paper,

- present quality of life (QOL) refers to the current life situation,

- change in quality of life since unemployment (CQOL) was measured only among unemployed.

In order to measure QOL the answers to the following question have been used: "How do you feel about your life as a whole just now?" This question had five response categories, scored from "very good" to "very bad". The other question focused on CQOL: "Do you think that your life as a whole has become better or worse since you became unemployed compared to directly before unemployment?" This question had five response categories, scored from "much better" to "much worse". According to Bowling [21], global and item-specific measures are appropriate for different research questions. The advantage of using a few global questions, rather than a whole battery, is the brevity. Our definition of QOL is in accordance with how Naess [16] and Bowling [18] defined it. The questions used in the questionnaire to measure QOL and CQOL have been used previously [20]. The questions have been chosen after pilot studies as items giving meaningful results, and have been followed by interviews that confirm the results. This indicates that the questions asked have high validity when used in order to shed light on a person's global QOL [20].

Variables showing a significant relation to QOL in a multiple analysis were measured by the following questions.

The symptoms anxiety, abdominal pain and myalgia/arthralgia were measured by the answers to the question "Have you been troubled by this symptom during the last month?" The answers were dichotomous: Yes or No.

Subjective health was measured by the answers to the question "How would you describe your overall health status at present?" with the five response categories "good, fairly good, neither good nor poor, fairly poor, poor".

Self-esteem was measured by Rosenberg's [22] self-esteem scale, which contains ten questions to measure self-esteem as a favourable or unfavourable attitude towards oneself. High self-esteem index indicates low self-esteem. Cronbach's α for the self-esteem index used was 0.71.

Economy and spare time were measured by the answers to the questions "How satisfied or dissatisfied are you with your economy" and "with your spare time?" with the five response categories "very satisfied, rather satisfied, neither satisfied nor dissatisfied, rather dissatisfied, very dissatisfied".

Opportunities to make one's own decisions were measured by the answers to the question "How are your possibilities to make your own decisions about your future?" with the five response categories "very good, good, neither good nor bad, bad, very bad".

### Statistical analyses

The significance of differences between proportions for nominal scale variables was tested by Fisher's exact test. Otherwise χ^2 ^test was used for qualitative variables.

Multivariate analyses were carried out by means of logistic regression models (method: enter) with QOL and CQOL as the dependent variables. QOL was dichotomised at the median into "good" (very good and rather good) and "not good" (neither good nor bad, rather bad and very bad). CQOL was dichotomised into "better" (much better and better) and "not better" (unchanged, worse and much worse). Explanatory variables included in the model were those with low correlation to each other (r_s _^2 ^< 0.20) and with a significant bivariate correlation to the dependent variable. Independent variables at ordinal scale or higher were first analysed as categorical in the logistic regression analyses. If they were not significantly related to QOL, they are presented as dichotomous instead. They were dichotomised as close as possible to the median value (Table 1). The reason for using the median to dichotomise the material is that we wanted a neutral split, free from subjective influence. This method has been used in a previous study [8]. Because of the salutogenetic approach in this study we used the concept Positive Odds Ratio (POR) instead of the commonly used odds ratio. The odds ratio was calculated in an ordinary way, but by changing positive and negative outcome in the dependent variable as well as in the explanatory variables [23].

**Table 1:** Variables included in the logistic regression with good/not good QOL as the dependent variable

**Variable**	**Scale**^a)^	**Categorised**
**Health-related variables**		
Dizziness	N	No/yes
Anxiety	N	No/yes
Difficulty in relaxing	N	No/yes
Overweight	N	No/yes
Lack of appetite	N	No/yes
Loss of weight	N	No/yes
Abdominal pain	N	No/yes
Headache	N	No/yes
Backache	N	No/yes
Myalgia/arthralgia	N	No/yes
Mental health index	Q: 0 to 6 symptoms	Low (0–2)/high (3–6)
Subjective health	O: Good (1) to poor (5)	Good (1)/rather good (2)/not good (3–5)
**Other variables**		
Relations to friends	O: Very satisfied (1) to very dissatisfied (5)	Satisfied (1)/not satisfied (2–5)
Close contact with persons outside home	N	Yes/no
Social contacts outside home	O: None (1) to >8 (4)	>8 persons (4)/8 persons or < 8 persons (1–3)
Feeling lonesome	O: Yes, often (1) to no, never (4)	No (3–4)/yes (1–2)
Support from parents/partner/friend/relatives	O: Very good support (1) to none (5) and not relevant (6) for each of these persons	Very good support (1) from at least one of these persons/less than very good support from all these persons (2–6)
Self-esteem index	Q: Low self-esteem index (0) to high self-esteem index (6)	Low (0)/high (1–6)
Satisfaction with:		
- economy	O: Very satisfied (1) to very dissatisfied (5)	Satisfied (1–3)/not satisfied (4–5)
- spare time	O: Very satisfied (1) to very dissatisfied (5)	Satisfied (1–2)/neither satisfied nor dissatisfied (3)/not satisfied (4–5)
- dwelling	O: Very satisfied (1) to very dissatisfied (5)	Satisfied (1–2)/not satisfied (3–5)
Opportunities to make own decisions	O: Very good (1) to very bad (5)	Very good (1)/good (2)/not good (3–5)
Married/cohabiting	N	No/yes
Living with parents	N	No/yes

a) N = Nominal scale; O = Ordinal scale; Q = Quote scale.

The significance level was set at 0.05. SPSS for Windows version 9.0 and EPI 5 (Epi info) version 5 were used for the statistical procedures.

The study was approved by the Committee on Ethics at the Faculty of Medicine, University of Lund (LU 309-98).

## Results

As can be seen from Table 2, the level of QOL was lower in unemployed males than in the reference group, principally because of different proportions of subjects who estimated their QOL as bad or neither good nor bad (30% compared to 10%). The difference in QOL between unemployed females and the reference group could mainly be explained by the smaller proportion that had estimated their QOL as very good (15% compared to 30%) and the larger proportion that had reported neither good nor bad QOL (24% compared to 10%).

**Table 2:** Present quality of life (QOL) in unemployed and in the reference group. Males and females

	**Males**			**Females**		
	Unemployed	Reference group	p-value	Unemployed	Reference group	p-value

QOL %	(n = 70)	(n = 168)		(n = 88)	(n = 189)	
Very good	19	33		15	30	
Rather good	51	57		51	55	
Neither good nor bad	17	8		24	10	
Bad ^a)^	13	2	0.000	10	5	0.001

a) Rather bad and very bad.

Among unemployed 24% felt that their QOL had become better (CQOL) and 35% estimated that QOL had become worse since unemployment started. A majority (67%) of individuals reported QOL as good. Of those individuals reporting their CQOL as worse, 36% (20/55) still reported good QOL (Table 3).

**Table 3:** Unemployed individuals' present quality of life (QOL), and change in quality of life (CQOL) since unemployment

**CQOL**				
**QOL**	Better^c)^	Unchanged	Worse^d)^	Total

Good ^a)^	34	50	20	104 (67%)
Neither/nor	3	12	18	33 (21%)
Bad^b)^	0	1	17	18 (12%)
Total	37 (24%)	63 (41%)	55 (35%)	155 (100%)

a) Very good and rather good.

b) Rather bad and very bad.

c) Much better and better.

d) Somewhat worse and much worse.

In the logistic regression models (Table 4), the health-related variables not having anxiety (POR = 4.10) and having good subjective health (POR = 11.46) were related to having good QOL among the unemployed. Other correlated variables were high self-esteem (POR = 4.22), and being content with spare time (POR = 16.52). Finally, having very good or good opportunities to make one's own decisions (POR = 11.62 and 4.62, respectively) were related to having good QOL among the unemployed.

**Table 4:** Positive odds ratios (POR) and 95% confidence intervals (CI) for variables related to good QOL. Results from logistic regression modelling of variables showing a bivariate correlation to QOL in unemployed and in the reference group

	**Unemployed **(n = 158)		**Reference group **(n = 355)	
**Health-related variables**	**POR**^a)^	**CI**	**POR**^a)^	**CI**
Dizziness: no	0.46	(0.12–1.75)	1.81	(0.75–4.37)
Anxiety: no	**4.10**	(1.21–13.96)	2.05	(0.80–5.28)
Difficulty in relaxing: no	1.20	(0.44–3.33)	1.15	(0.48–2.78)
Overweight: no	1.80	(0.70–4.64)	1.38	(0.53–3.57)
Lack of appetite: no	1.75	(0.55–5.61)	2.31	(0.84–6.34)
Loss of weight. no	1.56	(0.28–8.79)	0.96	(0.23–4.08)
Abdominal pain: no	**0.16**	(0.05–0.53)	1.00	(0.41–2.42)
Headache: no	1.77	(0.66–4.77)	1.02	(0.45–2.33)
Backache: no	0.68	(0.23–2.00)	2.08	(0.91–4.74)
Myalgia/arthralgia: no	1.34	(0.46–3.93)	**0.37**	(0.14–0.95)
Mental health index: low	1.20	(0.41–3.51)	2.70	(1.00–7.34)
Subjective health:				
not good (reference category)	1.00		1.00	
rather good	2.76	(0.88–8.64)	**4.32**	(1.76–10.60)
good	**11.46**	(3.36–39.06)	**11.91**	(4.00–35.44)
	(n = 149)		(n = 341)	
**Other variables**				
Relations to friends: satisfied	1.19	(0.37–3.76)	1.22	(0.50–3.16)
Close contacts with persons outside home: yes	1.50	(0.32–7.05)	0.86	(0.23–3.23)
Social contacts outside home: >8 persons	0.66	(0.22–1.93)	1.33	(0.58–3.07)
Feeling lonesome: no	1.95	(0.67–5.66)	1.87	(0.76–4.57)
Support from parents, partner, any friend or any relatives: very good	0.58	(0.14–2.34)	0.77	(0.22–2.62)
Self-esteem index: low (high self-esteem)	**4.22**	(1.26–14.05)	2.31	(0.70–7.59)
Economy: satisfied	2.10	(0.71–6.19)	**3.80**	(1.68–8.57)
Spare time:				
not satisfied (reference category)	1.00		1.00	
neither satisfied nor dissatisfied	4.76	(0.91–24.91)	1.86	(0.65–5.33)
satisfied	**16.52**	(3.03–90.12)	**6.64**	(2.27–19.43)
Dwelling: satisfied	1.51	(0.55–4.15)	1.70	(0.73–3.95)
Opportunities to make own decisions:				
not good (reference category)	1.00		1.00	
good	**4.62**	(1.47–14.61)	2.25	(0.88–5.78)
very good	**11.62**	(2.67–50.67)	1.98	(0.66–5.99)
Married/cohabiting: no	2.54	(0.65–9.98)	0.98	(0.37–2.61)
Living with parents: no	1.63	(0.55–4.83)	1.54	(0.55–4.36)

a) Positive Odds Ratios in bold when significant (p < 0.05).

Variables not showing a bivariate relation to QOL and therefore not included in the logistic regression models were: sex, education, living alone, living with children, immigration, exhaustion and breathlessness. The variables numbness/pricking in arms and legs and living with someone else consisted of too few individuals, and were therefore excluded from the logistic regression model.

In the reference group, having good or rather good subjective health (POR = 11.91 and 4.32, respectively), being content with one's economy (POR = 3.80), and being content with spare time (POR = 6.64) were related to having good QOL.

In a logistic regression model with CQOL as the dependent variable, two variables appeared to be related to having better QOL since unemployment started (CQOL). They were high self-esteem (POR = 3.17, CI: 1.22–8.21) and having more (5–8 and >8) social contacts outside the family (POR = 3.72, CI: 1.02–13.61 and POR = 6.05, CI: 1.81–20.18, respectively).

## Discussion

The main conclusion drawn from this study is that the picture of the unemployed young adults is diverse. As a group they experienced a lower QOL than those working or studying. On the other hand, a large number of young adults considered they had attained a better QOL since unemployment started (CQOL). Experiences of positive CQOL were related to high self-esteem and to more social contacts outside the home. According to our results reported in a previous paper [8], high self-esteem and good social support are related to better mental health, an important component in QOL, among unemployed young adults. The view that social support predicts levels of QOL [15] is supported in our study.

Our results balance the predominant picture of youth unemployment as a principally negative experience. The present study shows that unemployed young adults did report lower levels of QOL than the reference group. However, as many as two out of three (67%) unemployed young adults reported a good QOL (Table 3). And, perhaps most important, one out of four (24%) reported that QOL had become better and 41% reported that QOL was unchanged since unemployment started.

The prevailing view of unemployment is that those affected will suffer from it. It is well known that adolescents are hit by negative consequences of unemployment. For instance, it has been shown that mental health problems are over-represented among unemployed youth [5,8,24,25]. But it has also been suggested that negative effects are often relatively short-lived, and that unemployment is usually associated with a period of temporary discomfort or unhappiness [2]. Furthermore, it has been shown that unemployment produces clearly harmful effects, but at the same time there is a great variability in the subjective experience of unemployment [3,26].

As far as QOL is concerned, our results indicate that unemployment for a group of adults has a positive dimension too. Since QOL is here referred to as the individuals' evaluation of their life contents, i.e. their global QOL, it seems that many of the unemployed deal quite well with their situation as unemployed. We can here see a parallel to the results of a recent study [27] implying that unemployed young people (18–24 years) in the Nordic countries and Scotland managed unemployment well as regards coping patterns.

Several variables that are related to QOL among young people are highlighted in this study (Table 4). The fact that subjective health is strongly related to QOL [17,18] is confirmed. The relation is quite similar independent of employment status.

Being content with spare time appears to be as important for QOL among unemployed as for the reference group. It might be that the significance of being content with spare time is different between those groups. In the study by Julkunen [27] it was shown that being young and unemployed increased the time available for family, friends and hobbies, and being unemployed was also related to freedom in the use of time. However, being content with spare time can depend on how the spare time is used. It has been shown that unemployed young people spend more of their spare time doing nothing in particular compared to those who are employed, and that a purposeful use of spare time improves psychological well-being, both among unemployed and among those who are unsatisfactorily employed [28]. Regarding young people who work or study, it is likely that they experience less available time for family, friends and hobbies and also less freedom in the use of time. Even if satisfaction with spare time might be of differing significance, it is an important factor for QOL among the young adults in both the unemployed group and the reference group.

The ability to make one's own decisions was important for the QOL among unemployed; a sense of good opportunity to make one's own decisions and thus increased personal control is related to higher levels of QOL. It has been suggested that feelings of loss of control and decrease in feelings of personal efficacy accompany unemployment and increase the levels of distress [29]. It has also been shown that perceived control predicts subjective QOL among adolescents [15]. In the present study, it was shown that high self-esteem, i.e. "a favourable attitude towards oneself" [22] and low anxiety are related to higher levels of QOL among unemployed young people. It seems reasonable that individuals with high self-esteem and low anxiety experience life events more positively and thereby rate their QOL higher, a relation that is probably reciprocal. A central question, which is impossible to answer in this study, is whether correlates of unemployment are exposure or selection effects. Lower (or higher) well-being may depend on the negative (or positive) effects of unemployment (exposure), or it may be that individuals with low well-being become unemployed (selection). According to Hammarström and Janlert [30], there is support for both hypotheses when explaining the associations between unemployment and psychological ill health.

A feeling of having ability to make one's own decisions and having high self-esteem are important constituents of the concept of empowerment. Empowerment, aiming at increasing individuals' control over their lives, is essential in promoting health and QOL [31]. It has been suggested that governmental training programmes that enforce empowerment improve self-rated health and QOL among the unemployed [32]. Therefore, it could be expected that interventions which enhance feelings of personal control could reduce psychological and social distress among the unemployed. It seems relevant that personnel who deal with unemployed people make efforts aiming at empowering the unemployed by identifying their concerns, focusing on their resources. For this, individual treatment is required. One way might be to plan individual programmes in relation to personal development, education and work.

Being content with one's economic situation was related to higher levels of QOL in the reference group. A finding in the same direction, although not significant, was found among the unemployed. A Danish study suggests that assets like money, status and work do not seem to be important to one's QOL when unemployed [17]. One explanation for the modest relation between the economic situation and QOL among the unemployed in our study might be that their financial situation was not too bad. According to our results reported in a previous paper [8], more unemployed than studying or working young adults lived with their parents, a situation that might include parental support against economic shortcomings, and make the economic problems more manageable. Another explanation might be that the unemployed were not so discontented with their economic situation, because of the comparatively high benefit rates in Sweden. In a study by Hammer [10] it was shown that approximately 36% of the unemployed youth (age 18–24) in the Nordic countries reported no financial problems at all.

Jahoda's [11] theory, which is based more on the psychological needs that a "good" job should fulfil rather than the specific characteristics of unemployment, is often used to understand the consequences of unemployment. As most jobs fulfil such psychological needs, unemployment might be associated with lack of them. Our results reveal that QOL can be good even when these needs are not satisfied by employment, which indicates that young unemployed people manage to fulfil psychological needs in other ways. A similar conclusion was drawn by Nordenmark [14], who stated that unemployed people who manage to fulfil the psychosocial and the economic needs in other ways than paid employment feel rather well mentally. It has also been shown that unemployed individuals who have a proactive behaviour, i.e. being positive, creative and active, manage their unemployment situation quite well [33].

Even if we live in a society which emphasises the high societal and individual value of employment, a concept that is particularly obvious in Jahoda's [11] theory, it is questionable whether young people value employment in a corresponding way. According to our results, a great number of unemployed young adults reported good QOL and only a minority (35%) that QOL had become worse since unemployment started. It could thus be assumed that many young people value employment with regard to other criteria than those described by Jahoda.

Ezzy's [13] theory conceptualises unemployment as a type of status passage. In this respect, the consequences of becoming unemployed depend on how well the unemployed individual can keep a positive self-image, and a social identity, without a paid job. However, our results imply a very diversified picture of young adult's situation as unemployed and therefore the consequences of becoming unemployed may be assumed to vary. Our results show that a majority of unemployed young adults had reported that QOL was unchanged or even better than before unemployment. It is thus likely that most young adults manage this divestment passage rather well.

A limitation of this study is that this is a cross-sectional survey, which makes causal interpretations hazardous. Therefore we refer to relations between variables instead of emphasising the concept determinants. The methodological advantages of our study are primarily related to the fact that it is population-based, and that we compared with a randomly selected reference group. The response rates, 73% in unemployed and 71% in the reference group, were relatively high and a non-response analysis showed numerically small differences among responders and non-responders among unemployed as well as in the reference group. Using the concept of POR [23], i.e. looking at positive outcomes instead of negative ones, has made it possible to adapt the salutogenetic perspective to theories of unemployment.

## Conclusion

The general picture according to our results is that QOL is good among a majority of unemployed young adults. The relation between positive QOL and good health supports the conclusion that special attention should be paid to individuals who report reduced subjective health, especially anxiety, in order to achieve more equity in health. Positive QOL also seems to be related to high self-esteem, satisfaction with spare time and high space of decision-making. Therefore, efforts should aim at empowering unemployed young adults by identifying their concerns and resources. Suggestible, it can be done by creating individual programmes not only in relation to education and work, but also to personal development.

## Competing interests

The author(s) declare that they have no competing interests.

## Authors' contributions

LA participated in conceiving the study, carrying out the study and had the main responsibility for writing the manuscript.

IA participated in conceiving the study and writing the manuscript.

LE participated in conceiving the study and writing the manuscript.

GE participated in conceiving the study and writing the manuscript.

All authors read and approved the final manuscript.
